# Emetine induces oxidative stress, cell differentiation and NF-κB inhibition, suppressing AML stem/progenitor cells

**DOI:** 10.1038/s41420-024-01967-8

**Published:** 2024-04-29

**Authors:** Suellen L. R. Silva, Ingrid R. S. B. Dias, Ana Carolina B. da C. Rodrigues, Rafaela G. A. Costa, Maiara de S. Oliveira, Gabriela A. da C. Barbosa, Milena B. P. Soares, Rosane B. Dias, Ludmila F. Valverde, Clarissa A. G. Rocha, Nainita Roy, Christopher Y. Park, Daniel P. Bezerra

**Affiliations:** 1 Gonçalo Moniz Institute, Oswaldo Cruz Foundation (IGM-FIOCRUZ/BA), Salvador, BA 40296-710 Brazil; 2 SENAI Institute for Innovation in Advanced Health Systems, SENAI CIMATEC, Salvador, BA 41650-010 Brazil; 3 Department of Propaedeutics, Federal University of Bahia (UFBA), Salvador, BA 40301-155 Brazil; 4 Center for Biotechnology and Cell Therapy, D’Or Institute for Research and Education (IDOR), Salvador, BA 41253-190 Brazil; 5 Department of Pathology, School of Medicine, New York University, New York, NY 10016 United States of America

**Keywords:** Cancer stem cells, Acute myeloid leukaemia, Pharmacology

## Abstract

Acute myeloid leukemia (AML) is a fatal malignancy of the blood and bone marrow. Leukemic stem cells (LSCs) are a rare subset of leukemic cells that promote the development and progression of AML, and eradication of LSCs is critical for effective control of this disease. Emetine is an FDA-approved antiparasitic drug with antitumor properties; however, little is known about its potential against LSCs. Herein, we explored the antileukemic potential of emetine, focusing on its effects on AML stem/progenitor cells. Emetine exhibited potent cytotoxic activity both in hematologic and solid cancer cells and induced AML cell differentiation. Emetine also inhibited AML stem/progenitor cells, as evidenced by decreased expression of CD34, CD97, CD99, and CD123 in KG-1a cells, indicating anti-AML stem/progenitor cell activities. The administration of emetine at a dosage of 10 mg/kg for two weeks showed no significant toxicity and significantly reduced xenograft leukemic growth in vivo. NF-κB activation was reduced in emetine-treated KG-1a cells, as shown by reduced phospho-NF-κB p65 (S529) and nuclear NF-κB p65. DNA fragmentation, YO-PRO-1 staining, mitochondrial depolarization and increased levels of active caspase-3 and cleaved PARP (Asp214) were detected in emetine-treated KG-1a cells. Moreover, treatment with the pancaspase inhibitor Z-VAD(OMe)-FMK partially prevented the apoptotic cell death induced by emetine. Emetine treatment also increased cellular and mitochondrial reactive oxygen species, and emetine-induced apoptosis in KG-1a cells was partially prevented by the antioxidant *N*-acetylcysteine, indicating that emetine induces apoptosis, at least in part, by inducing oxidative stress. Overall, these studies indicate that emetine is a novel potential anti-AML agent with promising activity against stem/progenitor cells, encouraging the development of further studies aimed at its clinical application.

## Introduction

Acute myeloid leukemia (AML) is a heterogeneous disease exhibiting complex cytogenetic and molecular features and is characterized by the accumulation of immature myeloid progenitor cells, known as leukemic blasts, that disrupt the hematopoietic marrow and prevent normal blood cell production [1–3]. In the United States of America, 20,380 new AML cases were expected to occur by 2023, with approximately 11,310 AML fatalities. For AML patients aged 20 years and older, the 5-year survival rate is only 28%, underscoring the significant challenges associated with treating this disease [4].

Although there have been significant advances in our understanding of the biology of AML in recent years, induction therapy has remained mostly unchanged in recent decades and comprises cytarabine (ara-C) combined with an anthracycline (e.g. daunorubicin or idarubicin) [5]. Therefore, there is an urgent need to identify novel therapeutic agents to address this unmet medical need.

AML is initiated and maintained by a rare fraction of blasts known as leukemic stem cells (LSCs). These cells are capable of self-renewal and differentiation into leukemic progenitor cells and are therefore thought to be responsible for therapeutic resistance and disease recurrence. Recent studies have shown that control of this disease may be related to the ability of drugs to eliminate LSCs [6, 7]. Interestingly, some cellular and metabolic signaling pathways, including the NF-κB signaling pathway and oxidative stress pathways, have been studied as targets for eradicating LSCs [8–10].

**Fig. 1:**
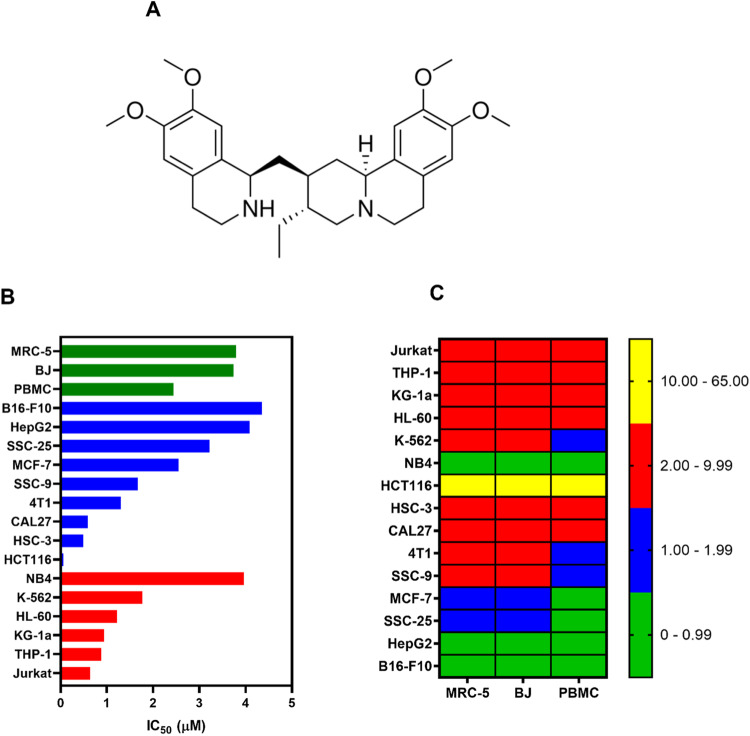
Emetine affects the viability of hematological and solid cancer cells. **A** Chemical structure of emetine. **B** IC_50_ values of the cytotoxicity of emetine against hematological (red bars) and solid cancers (blue bars), as well as against noncancerous cells (green bars). **C** Heatmap of selectivity indixes (SI) calculated for emetine using the following formula: SI = IC_50_ [noncancer cells]/IC_50_ [cancer cells].

Emetine (Fig. 1A) is a plant-derived isoquinoline alkaloid mainly isolated of *Cephaelis ipecacuanha*. It is an antiparasitic drug approved by the U.S. Food and Drug Administration (FDA) as a second-line agent for the treatment of protozoal infections [11]. Multiple pharmacological activities have been demonstrated for this molecule, including action against malaria [12], severe acute respiratory syndrome coronavirus 2 (SARS-CoV-2) [13], Zika virus [14] and Ebola virus [14].

Emetine has also been reported to exhibit cytotoxic effects against a variety of malignant tumors, including osteosarcoma [15], gastric cancer [16] and breast cancer [17]. In addition, some phase I or II clinical trials were performed with emetine in patients with solid tumors in the 1970s. Although some studies reported no benefit from emetine as a single agent, others observed disease stabilization, symptom relief, and reduction in tumors [18–22]. The anti-leukemic activity of emetine against AML has also been previously studied [23], but little is known about its potential against LSCs.

Emetine was discovered in a screen to identify inhibitors of NF-κB signaling using more than 2,800 clinically approved drugs [24]. Given that previous studies have implicated NF-kB signaling in LSC maintenance [8, 9] and that emetine has anti-AML potential [23], we hypothesized that emetine could eradicate AML stem/progenitor cells. Thus, we explored the anti-AML potential of emetine using a combination of in vitro and in vivo studies, focusing on its effects on AML stem/progenitor cells. We found that emetine inhibits AML stem/progenitor cells and that this action is associated with NF-κB inhibition and the induction of oxidative stress and cell differentiation.

## Results

### Emetine exhibits potent cytotoxicity to hematological and solid cancer cells

The cytotoxicity of emetine was assessed in six hematological cancer cell lines (KG-1a, HL-60, NB4, THP-1, Jurkat and K-562), nine solid cancer cell lines (MCF-7, 4T1, HCT116, B16-F10, HepG2, HSC-3, CAL27, SSC-9 and SSC-25), and three noncancerous cell lines (MRC-5, PBMCs, and BJ) by incubating the cells with emetine for 72 h, followed by the assessment of cell viability using the Alamar blue assay (Fig. 1B and Table S1).

The IC_50_ values of emetine for hematological cancers ranged from 0.64 µM for the human T-cell lymphoid leukemia Jurkat cell line to 3.96 µM for the human acute promyelocytic leukemia NB4 cell line. For solid cancers, the IC_50_ values ranged from 0.06 µM for the human colon cancer cell line HCT116 to 4.35 µM for the mouse melanoma B16-F10 cell line. For noncancerous cells, emetine had IC_50_ values of 3.79, 3.74, and 2.44 μM for MRC-5, BJ and PBMC cells, respectively. The selectivity index (SI) for each cell line was calculated using the following formula: SI = IC_50_ [noncancer cells]/IC_50_ [cancer cells] (Fig. 1C and Table S2). Emetine had an SI greater than two for most hematological cancer cells. Doxorubicin was used as a positive control and had IC_50_ values ranging from 0.02 µM for Jurkat cells to 1.53 µM for the mouse breast cancer 4T1 cell line. For noncancerous cells, doxorubicin exhibited IC_50_ values of 1.50, 3.23, and 1.31 μM for MRC-5, BJ and PBMC cells, respectively.

The AML cell line KG-1a has been shown to express hematopoietic stem/progenitor cell markers [25] and thus was selected for in vitro and in vivo studies to test the effects of emetine on AML stem-like cells. Emetine was tested at concentrations of 0.5, 1 and 2 µM based on the IC_50_ values for this cell line. To confirm the effect of emetine on KG-1a cell viability, viable cells were counted by the trypan blue exclusion method after 12, 24, 48, and 72 h of incubation (Figure S1). Emetine reduced the viability of KG-1a cells in a time-dependent manner, with cell viability reduced by 31.4 and 54.3% (0.5 µM), 53.4 and 66.2% (1 µM) and 56.6 and 75.9% (2 µM) after 12 and 24 h, respectively. After 48 and 72 h of treatment, cell viability was reduced by 72.3 and 88.6% (0.5 µM), 71.6 and 92.0% (1 µM), and 79.5 and 95.6% (2 µM), respectively.

### Emetine induces cell differentiation, suppresses AML stem/progenitor cells and exhibits antileukemic activity in a mouse xenograft model

**Fig. 2:**
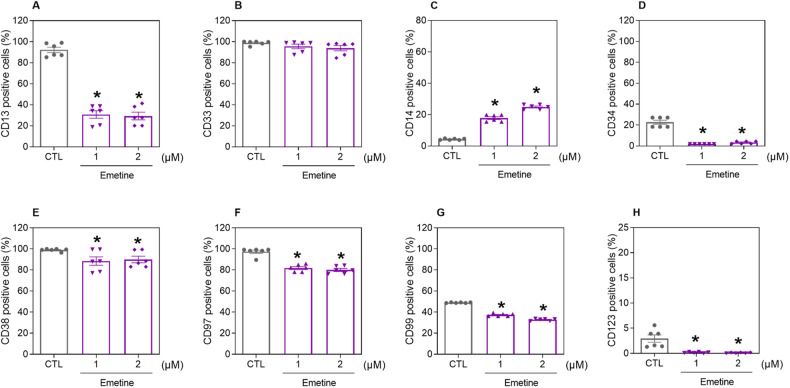
Emetine causes cell differentiation and suppresses AML stem/progenitor cells. Immunophenotypic analysis of the myeloid lineage markers CD13 (**A**) and CD33 (**B**), the AML cell differentiation marker CD14 (**C**), and the AML stem/progenitor markers CD34 (**D**), CD38 (**E**), CD97 (**F**), CD99 (**G**), and CD123 (**H**) was performed in emetine-treated KG-1a cells after 48 h of incubation. The vehicle (0.2% DMSO) was used as a negative control (CTL). The data are shown as the mean ± S.E.M. of three independent experiments carried out in duplicate. *p < 0.05 compared to CTL by one-way ANOVA followed by Dunnett’s multiple comparisons test.

Immunophenotypic analysis of the myeloid lineage markers CD13 and CD33, monocyte differentiation marker CD14, and AML stem/progenitor markers CD34, CD38, CD97, CD99, and CD123 was performed in emetine-treated KG-1a cells after 48 h (Fig. 2A–H). Emetine induced AML cell differentiation, as evidenced by increased CD14 expression, and decreased expression of the AML stem cell markers CD34, CD97, CD99, and CD123.

**Fig. 3:**
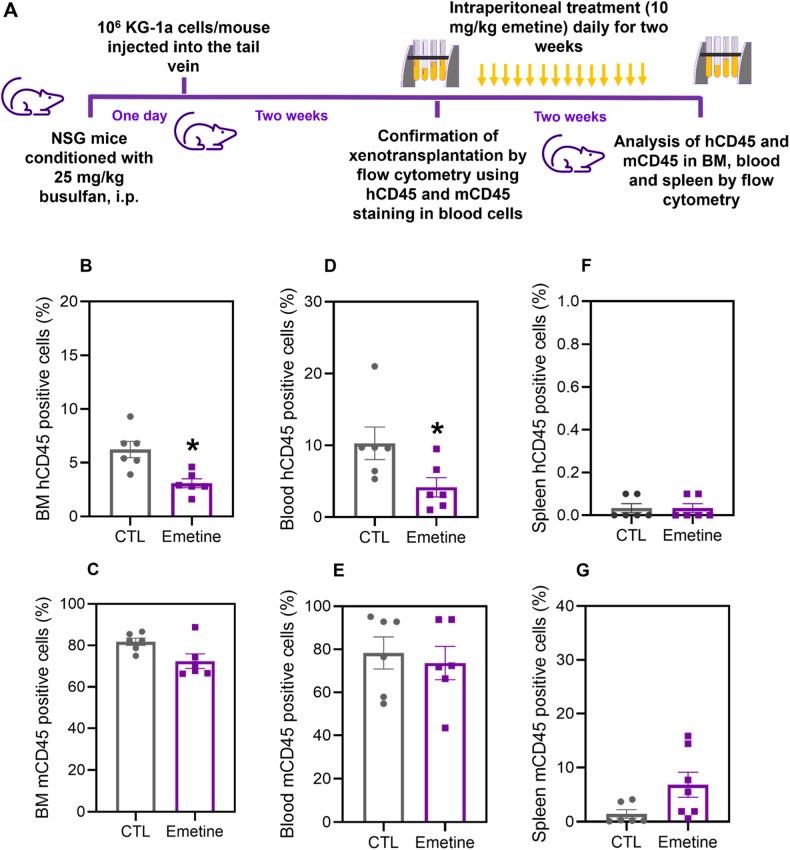
Effect of emetine on the growth of xenografts derived from KG-1a cells. **A** A xenograft model was established in NSG mice. Two weeks after the inoculation of KG-1a cells, the mice were randomly divided into the emetine (10 mg/kg) group and the control group (5% DMSO). hCD45-positive cells were quantified by flow cytometry from (**B**) bone marrow, (**D**) peripheral blood and (**F**) spleen. mCD45-positive cells were quantified by flow cytometry from (**C**) bone marrow, (**E**) peripheral blood, and (**G**) spleen. The data are shown as the mean ± S.E.M. of 6 animals. *p < 0.05 compared with CTL by Student’s *t* test.

To assess the in vivo antileukemic effect of emetine, an AML xenotransplantation model using KG-1a cells was used (Fig. 3A). Following transplantation of KG-1a cells and confirmation of engraftment, mice were treated with emetine (10 mg/kg) for two weeks, resulting in a significantly reduced leukemic burden in vivo (Fig. 3B–G). The percentage of engrafted human CD45-positive cells in the bone marrow and peripheral blood significantly decreased in the emetine-treated mice compared to the negative control group, while no change was observed in the spleen. No statistically significant differences were found in the percentage of CD45-positive mouse cells in emetine-treated mice, suggesting selectivity against leukemia engraftment.

No significant change in body weight or organ weight was found, with the exception of the spleen (Table S3). Histopathological examination of the kidneys, heart, lungs, and liver of emetine-treated mice revealed modest and/or reversible changes, suggesting little toxicity to normal tissues (Fig. S2).

### Emetine inhibits NF-κB signaling in AML cells

**Fig. 4:**
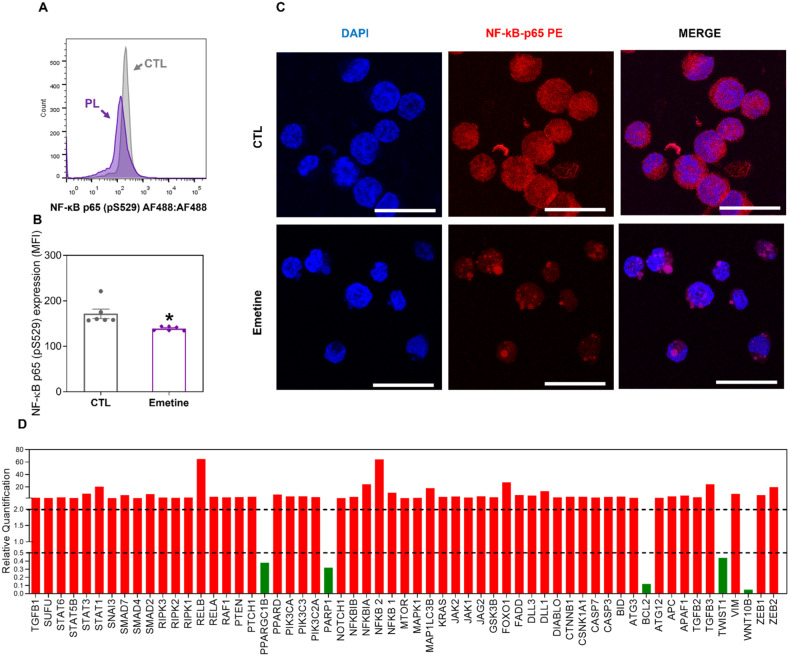
Emetine interferes with NF-κB signaling in KG-1a cells. **A**, **B** Effect of emetine on the levels of NF-κB p65 (pS529) after 24 h of treatment in KG-1a cells. The cells were treated with 2 μM emetine. The vehicle (0.2% DMSO) was used as a negative control (CTL). The data are shown as the mean ± S.E.M. of three independent experiments carried out in duplicate. *p < 0.05 compared with CTL by Student’s *t* test. MFI = mean fluorescence intensity. **C** Representative immunofluorescence images of NF-κB p65 in KG-1a cells after 24 h of incubation with 2 μM emetine. Scale bar = 25 μm. **D** Up- and downregulated genes in KG-1a cells after 12 h of treatment with 2 µM emetine. Genes that displayed RQ ≥ 2 (red bars) were upregulated, and RQs ≤ 0.5 (green bars) were downregulated.

Emetine has been previously reported to be an inhibitor of NF-κB signaling [24]. Therefore, we investigated the role of NF-κB in the effects of emetine on KG-1a cells. Both NF-κB phosphorylation and nuclear translocation are essential for its activity; thus, we quantified phospho-NF-κB p65 (S529) levels by flow cytometry and the localization of NF-κB p65 by confocal microscopy and observed that the KG-1a cell line has constitutive NF-κB activity. The levels of phospho-NF-κB p65 (S529) (Fig. 4A, B) were significantly reduced in the KG-1a cells treated with emetine. Decreased nuclear NF-κB p65 protein was also observed in emetine-treated KG-1a cells (Fig. 4C and Fig. S3), indicating that emetine inhibited NF-κB signaling in AML cells.

To better understand the molecular mechanism of action of emetine in KG-1a cells, we measured the relative expression of a panel of 92 genes via a qPCR array. This panel included important genes of the NF-κB, WNT/β-catenin, Hedgehog, NOTCH, EGFR, JAK/STAT, PI3K/AKT/MTOR, TGF-beta/SMAD and PPAR pathways, oxidative stress, apoptosis, autophagy, necroptosis and epithelial–mesenchymal transition. A total of 54 upregulated genes and five downregulated genes were detected in KG-1a cells following 2 μM emetine treatment for 12 h (Fig. 4D and Table S4), including the upregulation of NF-κB inhibitor genes (*NFKBIA*, RQ = 24.37 and *NFKBIB*, RQ = 4.05). Although the genes *NFKB1* (RQ = 10.59), *NFKB2* (RQ = 64.18), *RELA* (RQ = 4.36) and *RELB* (RQ = 64.73) were also upregulated, these data corroborate that emetine can interfere with NF-κB signaling. *WNT10B* (RQ = 0.05), *SUFU* (RQ = 2.33), *PTEN* (RQ = 3.62), *PPARGC1B* (RQ = 0.38), *APAF1* (RQ = 6.18), *CASP3* (RQ = 4.00), *CASP7* (RQ = 3.09), *MAP1LC3B* (RQ = 18.09), *RIPK3* (RQ = 3.15), and *TWIST1* (RQ = 0.44) were also among the genes whose expression was altered by emetine, indicating that this molecule may exert its cytotoxic effects by altering the transcription of multiple genes.

### Emetine induces oxidative stress-mediated apoptotic cell death in AML cells

**Fig. 5:**
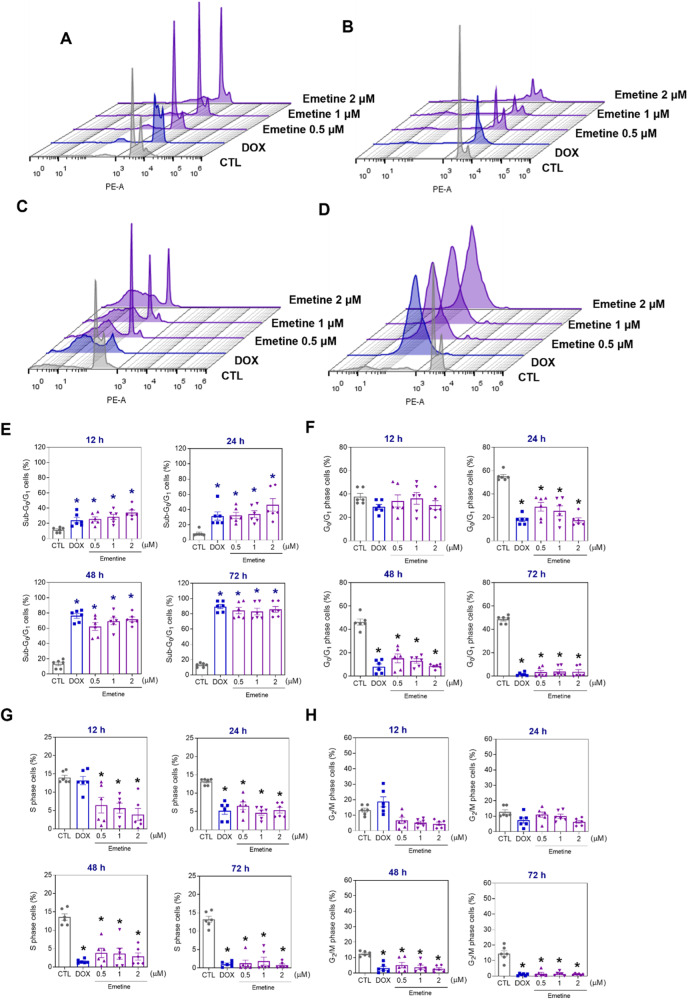
Cell cycle progression in KG-1a cells after incubation with emetine. Representative histograms after (**A**) 12, (**B**) 24, (**C**) 48, and (**D**) 72 h of treatment. Percentages of cells in (**E**) sub-G_0_/G_1_, (**F**) G_0_/G_1_, (**G**) S, and (**H**) G_2_/M after different incubation periods with emetine. Vehicle (0.2% DMSO) was used as a negative control (CTL), and doxorubicin (DOX, 1 µM) was used as a positive control. The data are shown as the mean ± S.E.M. of three independent experiments carried out in duplicate. *p < 0.05 compared with CTL by one-way ANOVA followed by Dunnett’s multiple comparisons test.

The mechanism underlying the cell death induced by emetine treatment was also investigated. Cellular DNA content was quantified in emetine-treated KG-1a cells to determine internucleosomal DNA fragmentation, as evidenced by the decrease in DNA content (<2n), and cell cycle progression after 12, 24, 48 and 72 h of treatment (Fig. 5A–H). Emetine treatment caused DNA fragmentation in a time-dependent manner. After 12 h and 24 h of treatment, DNA fragmentation was 25.7 and 32.3% (0.5 µM), 28.5 and 34.1% (1 µM), and 34.2 and 46.2% (2 µM) against the 11.5 and 8.9% detected in the control, respectively. DNA fragmentation of 62.3 and 84.3% (0.5 µM), 69.3 and 82.9% (1 µM), and 71.7 and 85.8% (2 µM) was detected after 48 and 72 h of treatment, respectively, while 13.3 and 12.8% DNA fragmentation was detected in the control. The percentage of cells in the G_0_/G_1_, S and G_2_/M cell cycle phases decreased proportionally after emetine treatment.

**Fig. 6:**
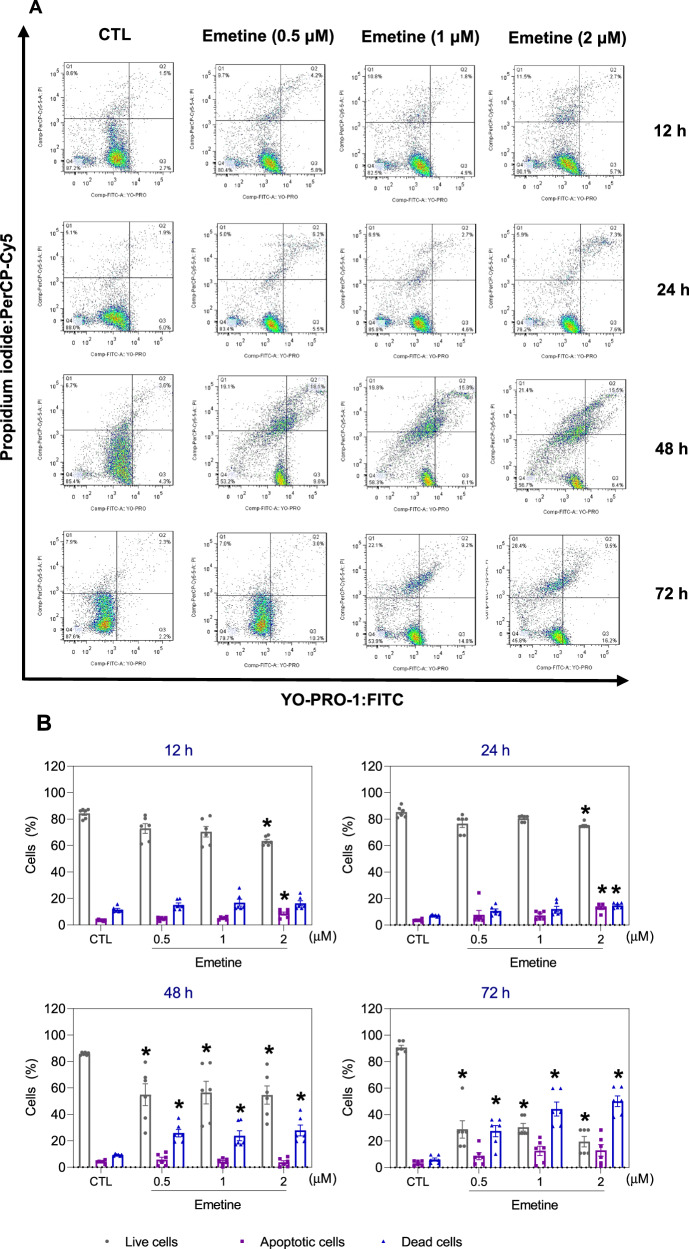
Apoptotic cell death induced by emetine in KG-1a cells. **A** Representative flow cytometry dot plots. **B** Apoptosis quantification in KG-1a cells after 12, 24, 48, and 72 h of treatment with emetine. Quantification of live (YO-PRO-1- and PI-double negative), apoptotic (YO-PRO-1-positive) and dead (YO-PRO-1- and PI-double positive) KG-1a cells. Vehicle (0.2% DMSO) was used as a negative control (CTL). The data are shown as the mean ± S.E.M. of three independent experiments carried out in duplicate. *p < 0.05 compared with CTL by one-way ANOVA followed by Dunnett’s multiple comparisons test.

The percentage of apoptotic emetine-treated KG-1a cells was quantified via YO-PRO-1/propidium iodide (PI) double staining by flow cytometry after 12, 24, 48, and 72 h (Fig. 6A, B). Emetine treatment increased the percentage of apoptotic cells and reduced the percentage of viable cells in a time-dependent manner. Cell shrinkage was also observed in the emetine-treated KG-1a cells, which was initiated after 24 h of incubation, as measured by a reduction in forward light scattering (Fig. S4).

**Fig. 7:**
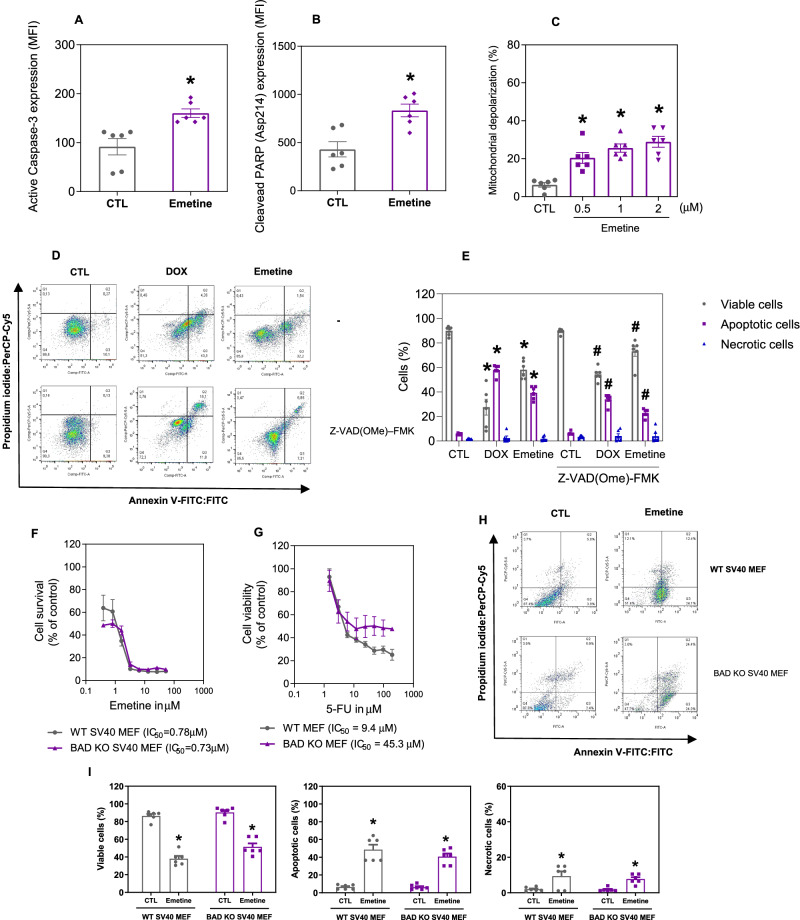
Emetine causes caspase-mediated apoptosis in KG-1a cells. **A** Effect of emetine on the levels of active caspase 3 and (**B**) cleaved PARP (Asp214) after 24 h of treatment in KG-1a cells. **C** Effect of emetine on mitochondrial activity in KG-1a cells. **D**, **E** Effect of the pancaspase inhibitor Z-VAD(OMe)-FMK on the apoptosis induced by emetine in KG-1a cells. The cells were pretreated for 2 h with 50 μM Z-VAD(OMe)-FMK and then incubated with 2 μM emetine for 48 h. **F**, **G** Survival curves of WT SV40 MEFs and BAD KO SV40 MEFs upon treatment with 5-fluorouracil (5-FU, a positive control) and emetine. The curves were obtained from at least three independent experiments carried out in duplicate using the Alamar blue assay after 72 h of incubation. **H**, **I** Induction of cell death in WT SV40 MEFs and BAD KO SV40 MEFs after 48 h of incubation with 2 μM emetine. Vehicle (0.2% DMSO) was used as a negative control (CTL), and doxorubicin (DOX, 1 µM) was used as a positive control. The data are shown as the mean ± S.E.M. of three independent experiments carried out in duplicate. *p < 0.05 compared with CTL by Student’s *t* test. ^#^*p* < 0.05 compared with the respective treatment without inhibitor by Student’s *t* test. MFI mean fluorescence intensity.

The induction of apoptosis by emetine was confirmed by the quantification of two apoptotic markers, active caspase-3 and cleaved PARP (Asp214), by flow cytometry. Emetine at 2 µM induced a significant increase in the level of active caspase-3 (Fig. 7A) and cleaved PARP (Asp214) (Fig. 7B) after 24 h. Mitochondrial dysfunction also participates in the induction of apoptosis, and the effects of emetine on mitochondrial membrane potential were assessed. Emetine increased the percentage of cells exhibiting mitochondrial depolarization (Fig. 7C). Finally, treatment with the pancaspase inhibitor Z-VAD(OMe)-FMK partially prevented the apoptotic cell death induced by emetine (Fig. 7D, E), indicating that emetine induces cell death, at least partially, through the induction of caspase-mediated apoptosis.

Next, the function of the BAD protein, a proapoptotic member of the Bcl-2 gene family, in emetine-induced cell death was evaluated. However, the effect of emetine appears to be independent of BAD, as it induces cell death in a similar manner in both BAD knockout mouse embryonic fibroblasts and their parental wild-type mouse embryonic fibroblasts (Fig. 7F–I).

**Fig. 8:**
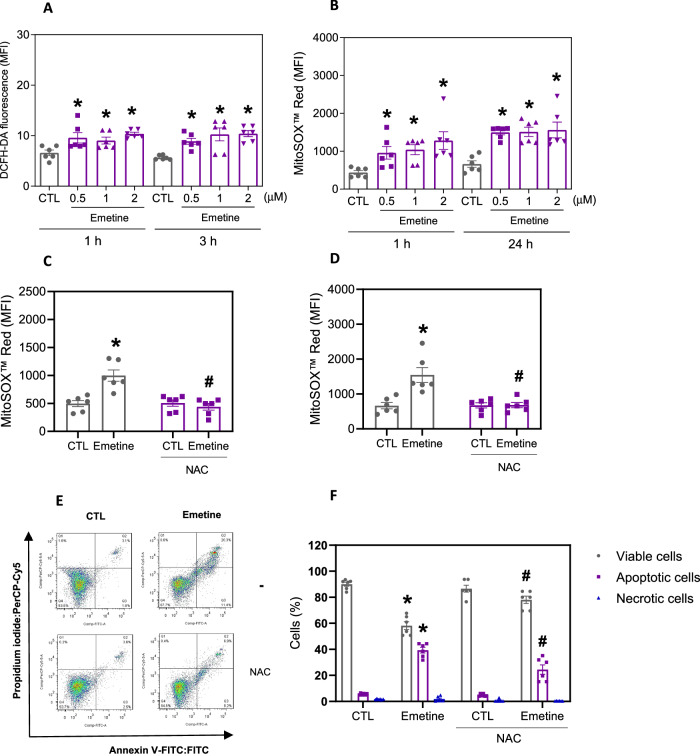
Induced oxidative stress in KG-1a cells by emetine. **A** Cellular ROS in KG-1a cells after 1 and 3 h of treatment with emetine. **B** Mitochondrial ROS in KG-1a cells after 1 and 24 h of treatment with emetine. Mitochondrial ROS in KG-1a cells after 1 (**C**) and 24 (**D**) h of treatment with 2 μM emetine pretreated with the antioxidant NAC. **E**, **F** Effect of the antioxidant NAC on the apoptosis induced by emetine in KG-1a cells. The cells were pretreated for 2 h with 5 mM NAC and then incubated with 2 μM emetine for 48 h. Vehicle (0.2% DMSO) was used as a negative control (CTL). The data are shown as the mean ± S.E.M. of three independent experiments carried out in duplicate. *p < 0.05 compared with CTL by Student’s *t* test or one-way ANOVA followed by Dunnett’s multiple comparisons test. ^#^*p* < 0.05 compared with the respective treatment without inhibitor by Student’s *t* test. MFI mean fluorescence intensity.

The role of oxidative stress in emetine-induced cell death was also investigated. First, we detected cellular and mitochondrial reactive oxygen species (ROS) in emetine-treated KG-1a cells stained with 2′,7′-dichlorofluorescin diacetate (DCF-DA) and MitoSOX, respectively. Emetine treatment increased both cellular and mitochondrial ROS (Fig. 8A, B), and mitochondrial ROS levels decreased when cells were pretreated with the antioxidant *N*-acetylcysteine (NAC) (Fig. 8C, D). In addition, NAC partially prevented the emetine-induced apoptosis in KG-1a cells, indicating that emetine induces apoptosis at least in part by inducing oxidative stress (Fig. 8E, F).

## Discussion

In this study, we demonstrated that the small molecule emetine exerts potent cytotoxic effects on hematologic and solid cancer cells and that this effect is associated with the induction of leukemia cell differentiation in culture, the suppression of AML stem/progenitor cells and the inhibition of leukemia development in a mouse xenograft model. Mechanistically, emetine interferes with NF-κB signaling and causes oxidative stress-mediated apoptotic cell death in AML cells.

The cytotoxicity of emetine has been previously investigated [15–17, 23]. Emetine reduced the clonogenic ability of AML cells without affecting hematopoietic stem cell function, synergized with ara-C cytotoxicity in AML cells, and reduced the development of AML HL-60 cells in NSG mice [23]. Emetine also induced cell death in glioblastoma [26] and breast cancer [27] cell lines enriched with cancer stem cells. Emetine also reduced the stem cell population in mucoepidermoid carcinoma cells and sensitized them to cisplatin [28]. However, the effects of emetine on LSCs have been almost unexplored. In the present work, we demonstrate for the first time that emetine can induce cell differentiation and suppress AML stem/progenitor KG-1a cells in vitro and their development in NSG mice.

Emetine has been shown to modulate multiple intracellular signaling pathways, inhibiting the Wnt/β-catenin, PI3K/AKT and Hippo/YAP signaling pathways in gastric cancer [16]; inhibiting Hedgehog signaling in an in-silico study [29]; and activating p38 and inhibiting ERK, JNK, and β-catenin signaling in osteosarcoma cells [15]. Emetine also reduced breast cancer cell survival by suppressing Wnt/β‑catenin signaling [17]. Emetine also inhibited NF-κB signaling in mucoepidermoid carcinoma cells [28]. Most of these intracellular signaling pathways are known targets in LSCs [9], and our studies demonstrated that emetine cytotoxicity and inhibition of stem cell marker expression are associated with interference with NF-κB signaling in KG-1a cells. This finding is not unexpected, as prior studies have demonstrated that NF-κB signaling is aberrantly active in AML bulk and AML stem cells but not in normal hematopoietic stem cells [8]. These findings indicate that it could be targeted selectively in LSCs.

Emetine also induced apoptosis in a caspase-dependent manner in KG-1a cells, like prior studies in cervical cancer cells [24] and pancreatic and bronchial cancer cells [30]. Indeed, emetine has previously been reported to induce apoptosis in various hematologic malignancies, including AML [23], chronic lymphocytic leukemia [31], and B-cell lymphoma [32].

We found that emetine increased ROS in KG-1a cells and that the cytotoxic effect of emetine could be prevented by the antioxidant NAC, indicating that oxidative stress induced by this molecule mediated this cell death mechanism. Emetine cytotoxicity could be prevented by the antioxidants L-ascorbic acid, L-cysteine, reduced glutathione, thiourea, deferoxamine, 1,3-dimethylthiourea and catalase in astrocytoma cells or by reduced glutathione in neuroblastoma cells, indicating an oxidative stress-mediated cell death mechanism [33]. Emetine has also been shown to induce pro-oxidative changes that disrupt the redox homeostasis of chronic lymphocytic leukemia cells [31]. In addition, AML cell differentiation is a ROS-mediated mechanism [34], which suggests that AML cell differentiation induced by emetine in KG-1a cells could be mediated by ROS. Together, these data support the effect of emetine on AML stem/progenitor cells, since oxidative stress has also been reported to be a molecular target for eliminating this subset of cancer cells [10].

These results indicate that emetine is a novel potential anti-AML agent with promising activity against stem/progenitor cells, encouraging the development of further studies aimed at its clinical application in cancer therapy.

## Materials and methods

### Emetine

Emetine was purchased from a commercial source (emetine dihydrochloride, #E2375, Sigma-Aldrich Co., Saint Louis, MO, USA).

### Cells

Table S5 details the cell lines used in this work, which included mouse and human cells. The cells were cultured according to the ATCC animal cell culture guidelines. All cell lines were grown in flasks at 37 °C in 5% CO_2_ and were replicated for 3–4 days to maintain exponential cellular growth. A 0.25% trypsin EDTA solution (Sigma-Aldrich Co., Saint Louis, MO, USA) was used to detach adherent cells. All cell lines were shown to be mycoplasma free by screening for mycoplasma using a mycoplasma staining kit (Sigma-Aldrich Co.). Viable cell counting was performed using the trypan blue exclusion method, and at the beginning of each experiment, the cells showed viability greater than 90%.

### Alamar blue assay

The Alamar blue assay was used to evaluate cell viability [35]. Briefly, the cells were seeded in 96-well culture plates (30,000 cells/well for suspension cells and 7,000 cells/well for adherent cells) and kept at 37 °C in 5% CO_2_. Emetine was added to each well, and the plates were incubated for 72 h in duplicate. Doxorubicin (purity ≥95%, Laboratory IMA S.A.I.C., Buenos Aires, Argentina) served as a positive control. Four hours before the end of the incubation period (or 24 h for PBMCs), resazurin was added to each well at a final concentration of 3 μM. The absorbance values at 570 nm and 600 nm were measured using a SpectraMax 190 Microplate Reader (Molecular Devices, Sunnyvale, CA, USA).

### Immunophenotyping assay

Phenotyping was carried out using two antibody panels. In the first panel, antibodies against CD13, CD33, CD34, CD38, and CD123 were used, and in the second panel, antibodies against CD14, CD97, and CD99 were used. Primary antibodies conjugated with specific fluorochromes were used and are detailed in Table S6. For both panels, the cells were washed with incubation buffer (0.5% bovine serum albumin in PBS) and incubated with antibodies for 1 h at room temperature. YO-PRO-1 or DAPI (Sigma-Aldrich Co.) was used to select viable cells. Then, the cells were washed with PBS, and cell fluorescence was determined by flow cytometry using a BD LSRFortessa cytometer and BD FACSDiva Software (BD Biosciences, San Jose, CA, USA) or FlowJo Software 10 (FlowJo LCC, Ashland, OR, USA). At least 30,000 events/sample were acquired. Cell doublets and debris were excluded from the analyses. The flow cytometry gating strategy is shown in Figs. S5 and S6.

### Xenotransplantation of leukemia cells

Twelve NOD. Cg-*Prkdc*^*scid*^ *Il2rg*^*tm1Wjl*^/SzJ (NSG) mice (male and female, 20–25 g) were obtained and housed under specific pathogen-free conditions by FIOCRUZ-BA animal facilities (Salvador, Bahia, Brazil). The experimental protocol was approved by a local animal ethics committee (#16/2018). All mice were fed a standard pellet diet (food and water available ad libitum) and housed in an artificially lit room (12 h dark/light cycle).

Recipients were conditioned with 25 mg/kg busulfan (Sigma-Aldrich Co.) one day before receiving KG-1a cells. On the following day, the animals were inoculated with 10^6^ cells/mouse via the tail vein. Mice were observed each day for signs of weight loss or lethargy. After two weeks, engraftment was confirmed in the peripheral blood by flow cytometry using both PE-conjugated anti-human CD45 (hCD45) and FITC-conjugated anti-mouse CD45 (mCD45) antibodies. Table S6 details all the antibodies used. The flow cytometry gating strategy is shown in Fig. S7.

After confirmation of engraftment, the animals were randomly divided into two groups (*n* = 6/per group): a negative control group (5% DMSO, vehicle) and an emetine-treated group (10 mg/kg). The animals were treated with the appropriate therapy every day for two weeks by intraperitoneal injection and then euthanized with an anesthetic overdose (thiopental, 100 mg/kg). Cells from the spleen, peripheral blood, and bone marrow were obtained, and the collected cells were analyzed by flow cytometry using PE-conjugated hCD45 and FITC-conjugated mCD45 double staining.

Kidneys, lungs, hearts, and livers were removed for toxicological evaluation. These organs were examined for color change, gross lesion formation, and/or bleeding, fixed in 4% formaldehyde, dehydrated through a graded alcohol series, washed in xylene, and embedded in paraffin wax. Tissues were cut into 5 μm thick slices, stained with hematoxylin-eosin and/or periodic acid-Schiff (liver and kidney), and examined under a light microscope.

### Gene expression analysis by qPCR array

KG-1a cells were incubated with 2 μM emetine for 12 h. Total RNA was isolated using the RNeasy Plus Mini Kit (Qiagen; Hilden, Germany) according to the manufacturer’s instructions. RNA purity was analyzed and quantified using a NanoDrop® 1000 spectrophotometer (Thermo Fisher Scientific, Waltham, MA, USA). RNA reverse transcription was performed using the Superscript VILO™ kit (Invitrogen Corporation; Waltham, MA, USA).

RT‒qPCR analysis was performed on a TaqMan® Array Plate 96 plus fast (#4413256, Applied Biosystems™, Foster City, CA, USA) on an ABI ViiA7 system (Applied Biosystems™). The PCR cycling conditions were 50 °C for 2 min and 95 °C for 10 min, followed by 40 cycles of 95 °C for 15 sec and 60 °C for 1 min. The relative quantification (RQ) of mRNA expression was calculated using the 2^-ΔΔCT^ method [36] with Gene Expression Suite™ software (Applied Biosystems™). Cells treated with 0.2% DMSO (negative control) were used for comparison. The geometric mean RQs of the three reference genes *GUSB*, *HPRT1* and *GAPDH* were used for data normalization. All experiments were performed under DNase/RNase-free conditions. A gene was upregulated if its RQ ≥ 2. Similarly, genes were downregulated when RQ ≤ 0.5.

### NF-κB p65 studies

For quantification of phospho-NF-κB p65 (S529), cells were harvested and resuspended in 0.5–1 mL of 4% formaldehyde for 10 min at 37 °C, followed by intracellular cell staining. Briefly, the tube was then placed on ice for 1 min. The cells were permeabilized on ice for 30 min by slowly adding ice-cold 100% methanol to prechilled cells with gentle vortexing until the final concentration of methanol reached 90%. After washing with incubation buffer (0.5% bovine serum albumin in PBS), primary antibodies conjugated with specific fluorochromes were added and incubated for 1 h at room temperature. Table S6 contains the details of the antibody used. Finally, the cells were rinsed with PBS, and cell fluorescence was analyzed by flow cytometry. At least 10,000 events were acquired per sample.

To examine the localization of NF-κB p65, cells were seeded into 24-well plates and exposed to 2 μM emetine for 24 h. After the incubation period, the cells were rinsed twice with PBS, plated as droplets (5 μL) on coverslips, permeabilized with Triton X-100 (0.5%), treated with RNase (10 μg/mL), rinsed with PBS, and incubated overnight with an anti-NF-κB p65 antibody (Table S6 contains antibody details). On the next day, the cells were rinsed with PBS and mounted with Fluoromount-G (Invitrogen, Thermo Fisher Scientific) containing DAPI. The cells were examined using a Leica TCS SP8 confocal microscope (Leica Microsystems, Wetzlar, HE, Germany).

### Internucleosomal DNA fragmentation and cell cycle progression

Internucleosomal DNA fragmentation and cell cycle progression were quantified by measuring DNA content via PI staining [37]. Briefly, the cells were permeabilized and stained with a solution containing 0.1% Triton X-100, 2 μg/mL PI, 0.1% sodium citrate and 100 μg/mL RNase (all from Sigma-Aldrich Co.). After a 15 min incubation in the dark, cell fluorescence was measured by flow cytometry as described above. At least 10,000 events were analyzed per sample.

### Apoptosis detection

For apoptosis quantification, cell viability was evaluated by flow cytometry using annexin V-FITC/PI (FITC Annexin V Apoptosis Detection Kit I, BD Biosciences) or YO-PRO-1/PI (Sigma-Aldrich Co.) according to the manufacturer’s instructions. At least 10,000 events/sample were acquired. For the functional assays, the antioxidant NAC and the pancaspase inhibitor Z-VAD(OMe)-FMK were used.

To detect the levels of cleaved PARP (Asp 214) and active caspase-3, the cells were analyzed by flow cytometry using a protocol for intracellular cell staining, as detailed above. At least 10,000 events were acquired per sample.

Mitochondrial transmembrane potentials were measured using the rhodamine 123 uptake method [38]. After 24 h of treatment, the cells were diluted with 1 μg/mL rhodamine solution (Sigma-Aldrich Co.) and incubated for 15 min at 37 °C in the dark. The cells were then centrifuged and resuspended in PBS. Cell fluorescence was analyzed by flow cytometry. At least 10,000 events were acquired per sample.

### ROS level analysis

Cellular ROS levels were measured using DCF-DA (Sigma-Aldrich Co.) by flow cytometry, as previously reported [39]. MitoSOX™ Red reagent (Thermo Fisher Scientific, Waltham, MA, USA) was used to detect mitochondrial ROS levels by flow cytometry, and analysis was performed according to the manufacturer’s instructions. At least 10,000 events were acquired per sample.

### Statistical analysis

The data are presented as the mean ± S.E.M. or as IC_50_ values with 95% confidence intervals from at least three independent biological replicates (done in duplicate). For statistical analysis, two-tailed unpaired Student’s *t* test (data from two groups) or one-way analysis of variance (ANOVA) followed by Dunnett’s multiple comparisons test (data from three or more groups) were performed using GraphPad Prism statistical software (Intuitive Software for Science; San Diego, CA, USA). *P* < 0.05 was considered significant.

### Supplementary information

Supplemental material

## Data Availability

Data will be made available on request.
