# Supplementary material for: Emetine induces oxidative stress, cell differentiation and NF-κB inhibition, suppressing AML stem/progenitor cells
Source: Cell Death Discov. 2024 Apr 29;10:201. doi: 10.1038/s41420-024-01967-8 (PMC11059384; doi:10.1038/s41420-024-01967-8)
Supplement: Supplementary file 1 — Supplemental material [file 41420_2024_1967_MOESM1_ESM.pdf]

Supplementary Material

Emetine induces oxidative stress, cell differentiation and NF-κB inhibition, suppressing AML stem/progenitor cells

Suellen L. R. Silva[1], Ingrid R. S. B. Dias[1], Ana Carolina B. da C. Rodrigues[1], Rafaela G. A. Costa[1], Maiara de S. Oliveira[1], Gabriela A. da C. Barbosa[1], Milena B. P. Soares[1,2], Rosane B. Dias[1,3], Ludmila F. Valverde[1], Clarissa A. G. Rocha[1,3,4], Nainita Roy[5], Christopher Y. Park[5], Daniel P. Bezerra[1,*,]

[1]Gonçalo Moniz Institute, Oswaldo Cruz Foundation (IGM-FIOCRUZ/BA), Salvador, BA, 40296-710, Brazil.

[2]SENAI Institute for Innovation in Advanced Health Systems, SENAI CIMATEC, Salvador, BA, 41650-010, Brazil.

[3]Department of Propaedeutics, Faculty of Dentistry, Federal University of Bahia (UFBA), Salvador, BA, 40301-155, Brazil.

[4]Center for Biotechnology and Cell Therapy, D'Or Institute for Research and Education (IDOR), Salvador, BA, 41253-190, Brazil.

[5]Department of Pathology, School of Medicine, New York University, New York, NY, 10016, United States of America.

*Corresponding authors:

D. P. Bezerra, E-mail: daniel.bezerra@fiocruz.br

Phone number: + 55 71 3176 2272.

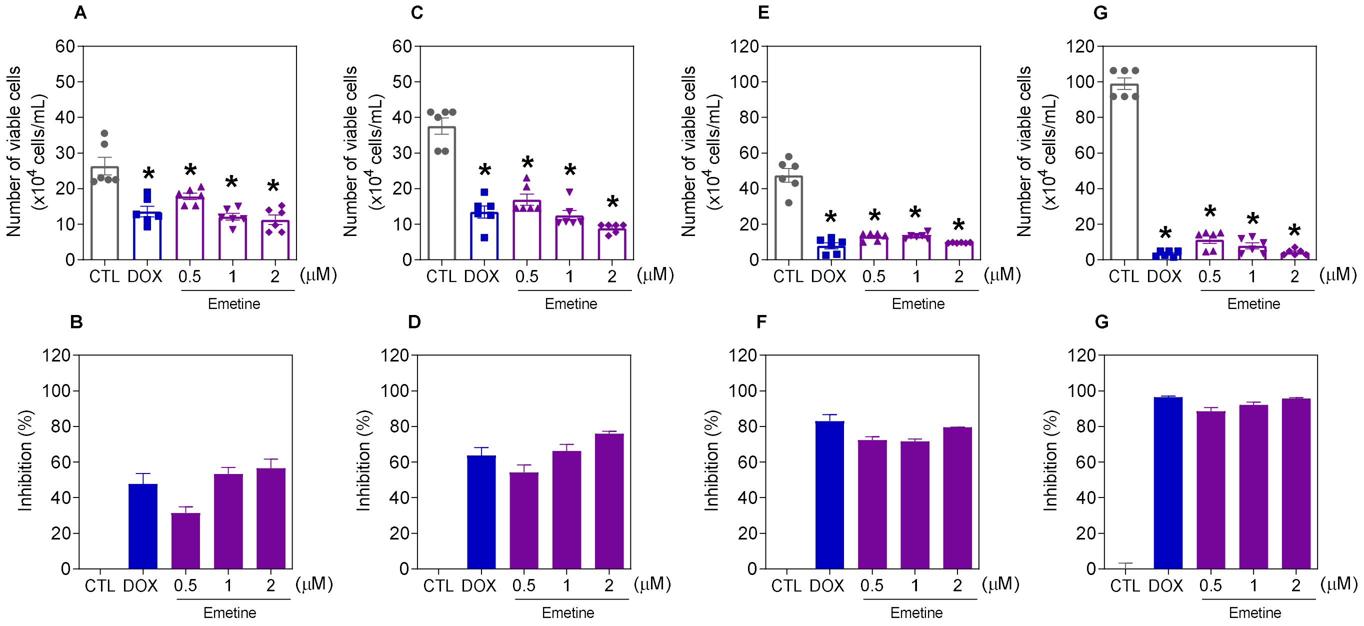

Figure S1. Trypan blue exclusion assay after (**A** and **B**) 12, (**C** and **D**) 24, (**E** and **F**) 48 and (**G** and **H**) 72 h of treatment with emetine in KG-1a cells. Vehicle (0.2% DMSO) was used as a negative control (CTL), and doxorubicin (DOX, 1 μM) was used as a positive control. The data are expressed as the mean ± S.E.M. of three independent experiments carried out in duplicate. * $p < 0.05$ compared with CTL by one-way ANOVA followed by Dunnett's multiple comparisons test.

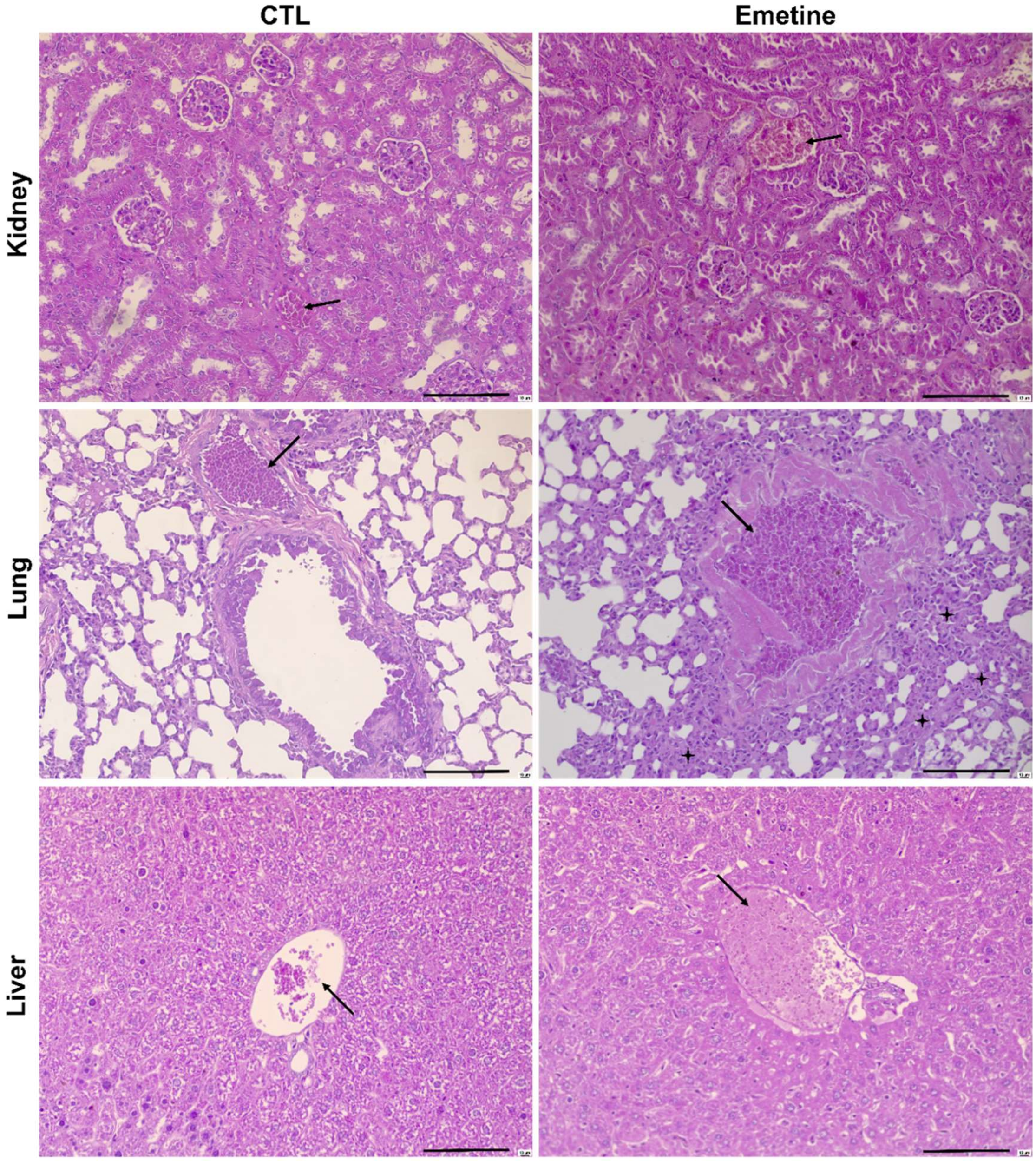

Figure S2. Representative photomicrographs of the kidneys, lungs, and livers of NOD. Cg-*Prkdc*^{scid} *Il2rg*^{tm1Wjl}/SzJ (NSG) mice with AML KG-1a cell xenografts that were treated with 10 mg/kg emetine. The negative control (CTL) was treated with a the vehicle (5% DMSO) used for dilution of emetine. The treatments were injected into the mice intraperitoneally every day for two weeks. The black arrows indicate vascular hyperemia. Scale bar = 100 µm. Histological analysis of various

organs (liver, lung, kidney, and heart) revealed histopathological changes in both the CTL and emetine experimental groups. The hearts of the experimental groups did not present architectural or morphological alterations (data not shown). In the livers, the architectures of the hepatic parenchyma and portal system ranged from preserved to partially altered in the CTL and emetine groups. The observed histopathological changes included vascular hyperemia, hydropic degeneration, and mixed tissue inflammation. These changes ranged from mild to severe, but events were more evident in the emetine-treated group. In addition, punctual areas of coagulation necrosis were observed in hepatocytes, mainly in regions close to the centrilobular vein. In the lungs, the architecture of the lung parenchyma was partially preserved in all animals in the CTL and emetine groups. Thickening of the alveolar septa with airspace atelectasis was observed in all animals (demonstrated by stars in the photomicrograph), ranging from mild to severe. Other histological alterations were observed in this organ, such as vascular hyperemia, edema, and inflammation with predominant polymorphonuclear infiltration. In addition, focal areas of hemorrhage were observed in the lungs of the animals. In the kidneys, the renal architecture was maintained in all experimental groups. In general, the histopathological changes observed in this organ were moderate vascular hyperemia and mild glomerular hyalinization with decreased urinary space in all groups. In addition, focal areas of coagulation necrosis in the renal cortex tubules and fibrosis were observed. Some histopathological features, such as hydropic degeneration, vascular congestion, and focal areas of inflammation, are acute cellular responses to treatment, and injured cells can return to homeostasis when aggression ends.

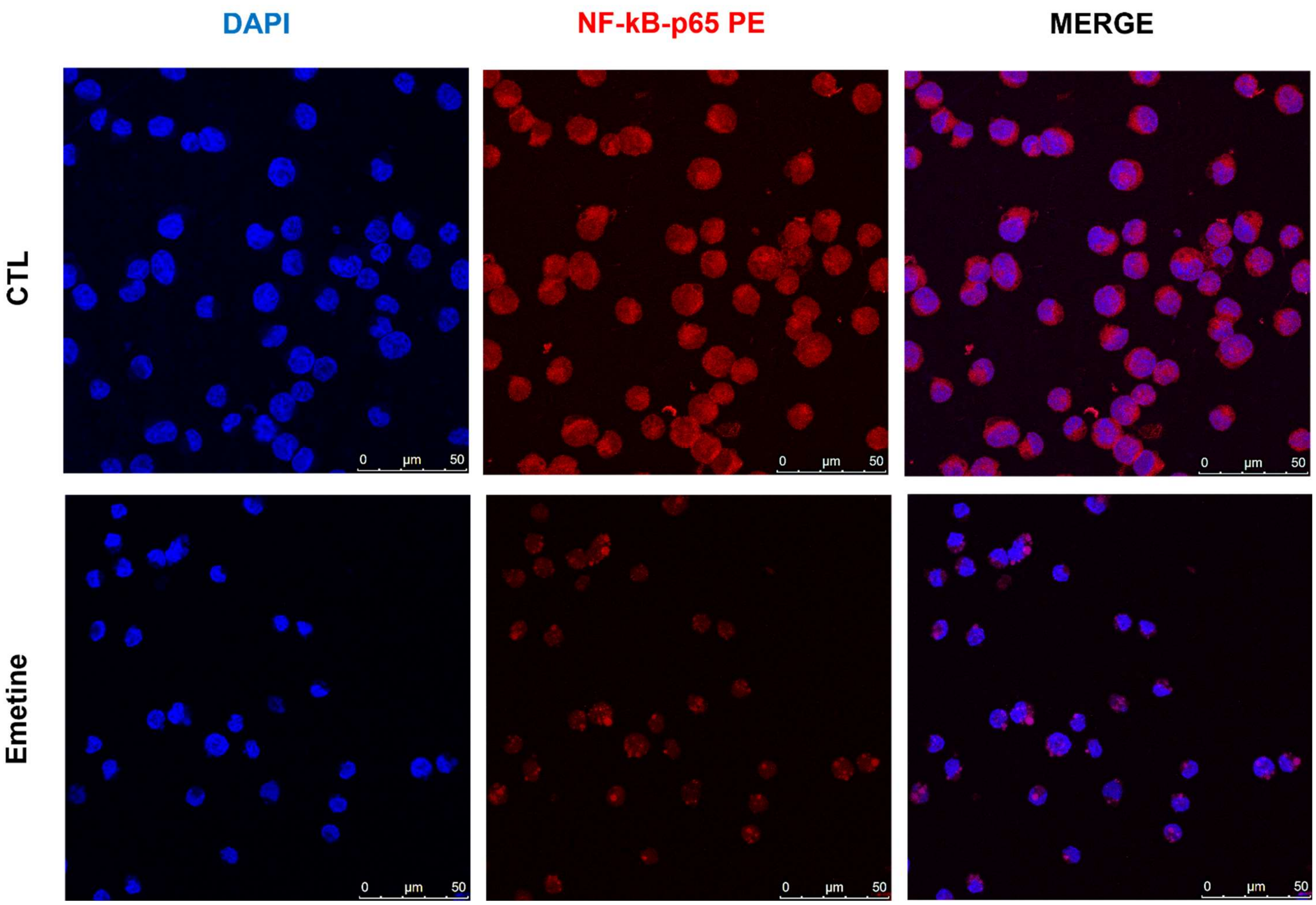

Figure S3. Uncropped immunofluorescence images of NF-κB p65 in KG-1a cells after 24 h of incubation with 2 μM emetine (as shown in Figure 4C). Scale bar = 50 μm.

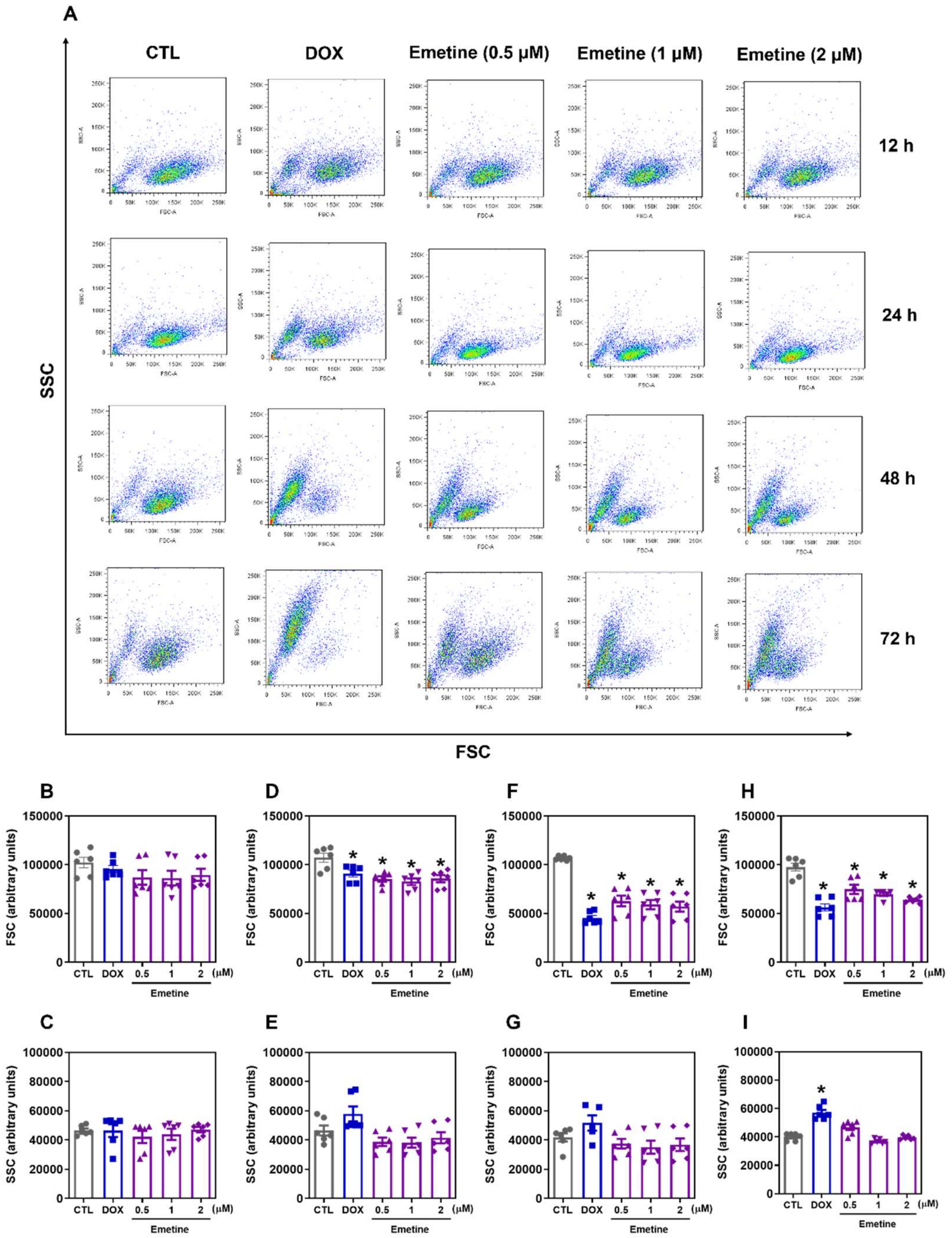

Figure S4. Effect of emetine on the morphology of KG-1a cells, as determined by light-scattering features (forward scatter – FSC and e side scatter – SSC) detected by flow cytometry after 12 (**A, B** and **C**), 24 (**A, D** and **E**), 48 (**A, F** and **G**), and 72 (**A, H** and **I**) h of incubation. The vehicle (0.2% DMSO) was used as a control (CTL), and doxorubicin (DOX, 1 μM) was used as a positive control. The data are shown as the mean ± S.E.M. of three independent experiments carried out in duplicate. * $p < 0.05$ compared to CTL by one-way ANOVA followed by Dunnett's multiple comparisons test.

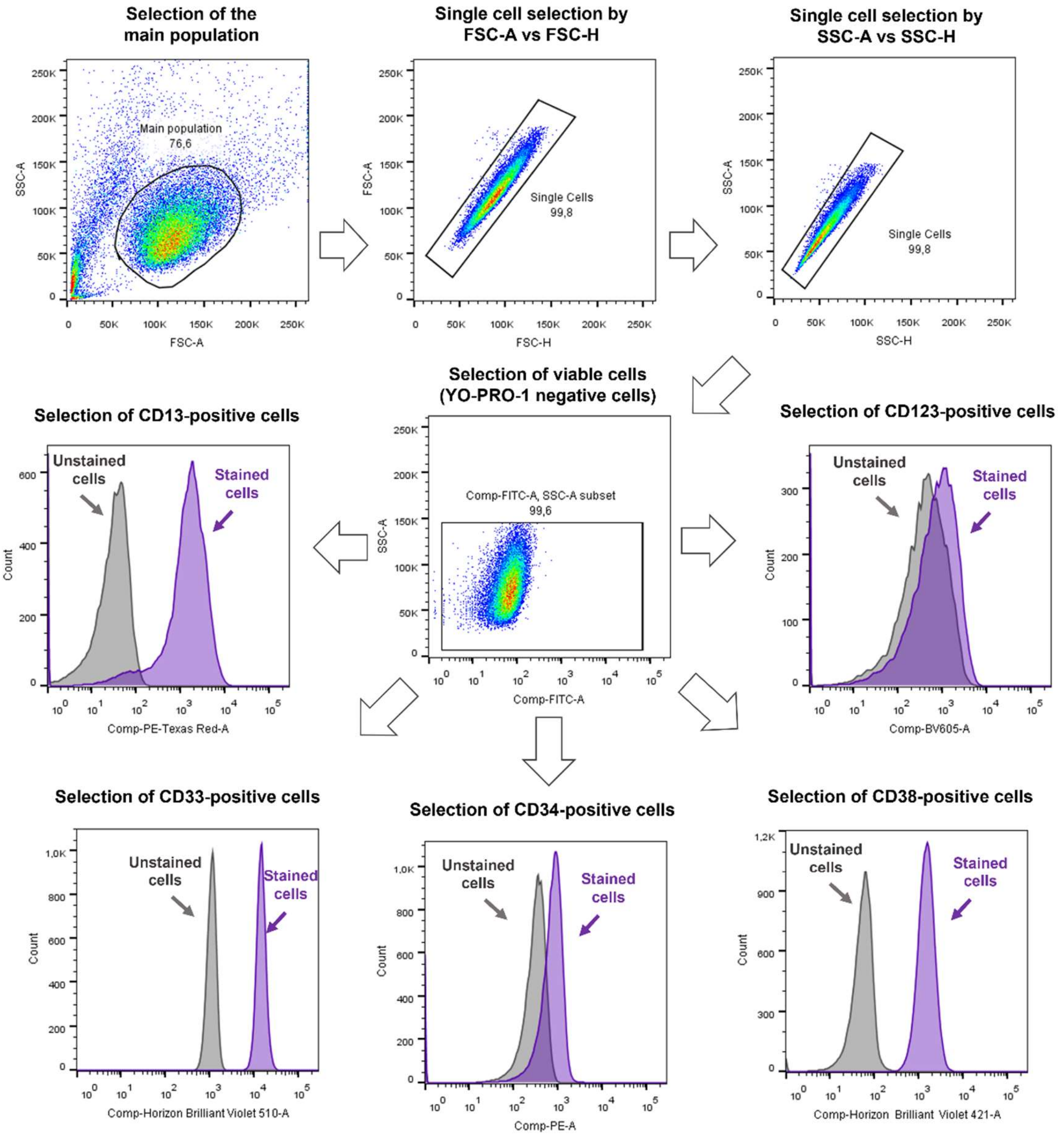

Figure S5. Representative gating strategy used for immunophenotyping of KG-1a cells via flow cytometry for panel 1. PE-CF594-conjugated mouse anti-human CD13, BV510-conjugated mouse anti-human CD33, PE-conjugated mouse anti-human CD34, BV421-conjugated mouse anti-human CD38, and BV605-conjugated mouse anti-human CD123 antibodies were used. YO-PRO-1 was used to select viable cells.

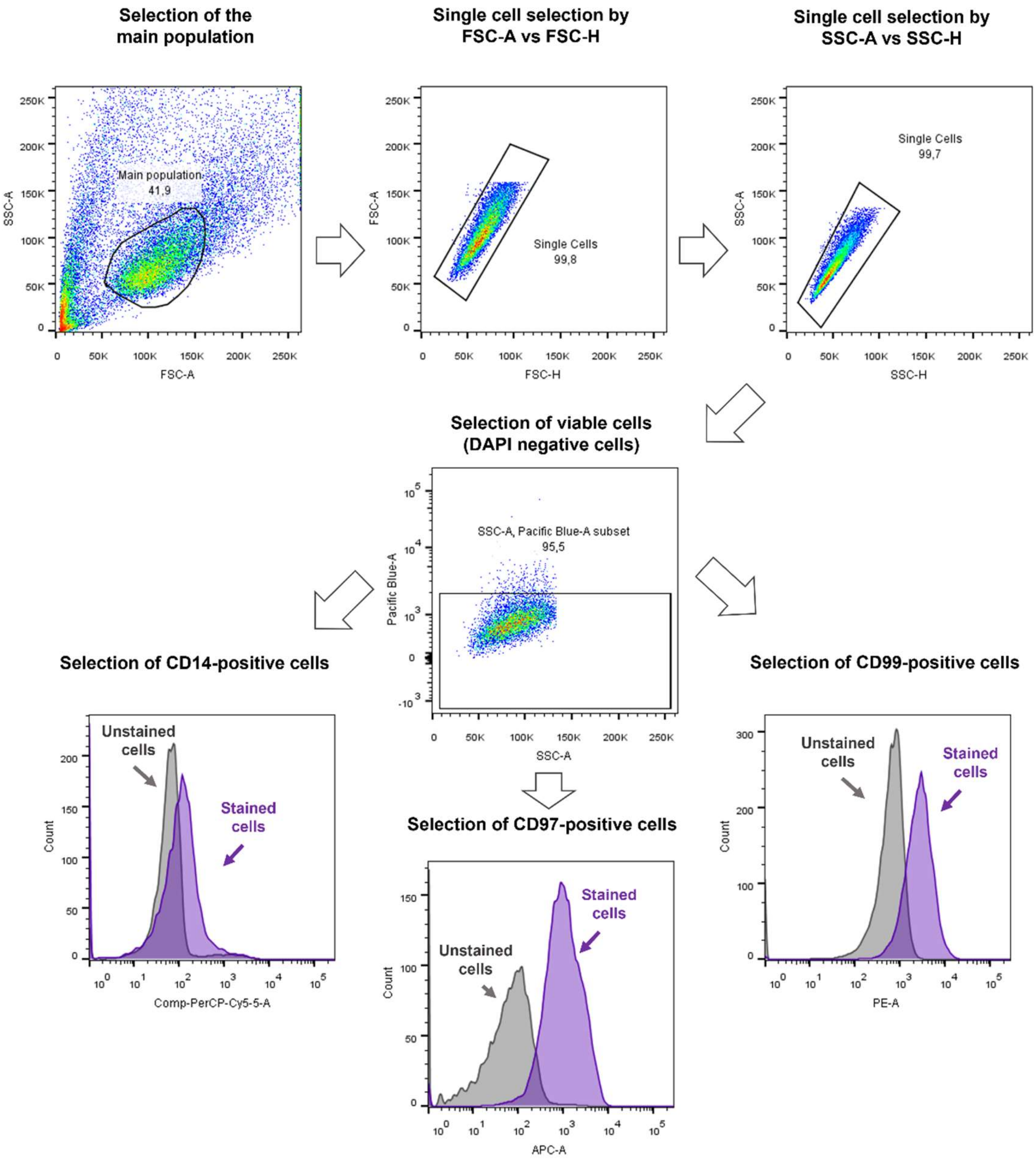

Figure S6. Representative gating strategy used for immunophenotyping of KG-1a cells via flow cytometry for panel 2. PerCP-Cy5-conjugated mouse anti-human CD14, APC-conjugated mouse anti-human CD97 and PE-conjugated mouse anti-human CD99 antibodies were used. DAPI was used to select viable cells.

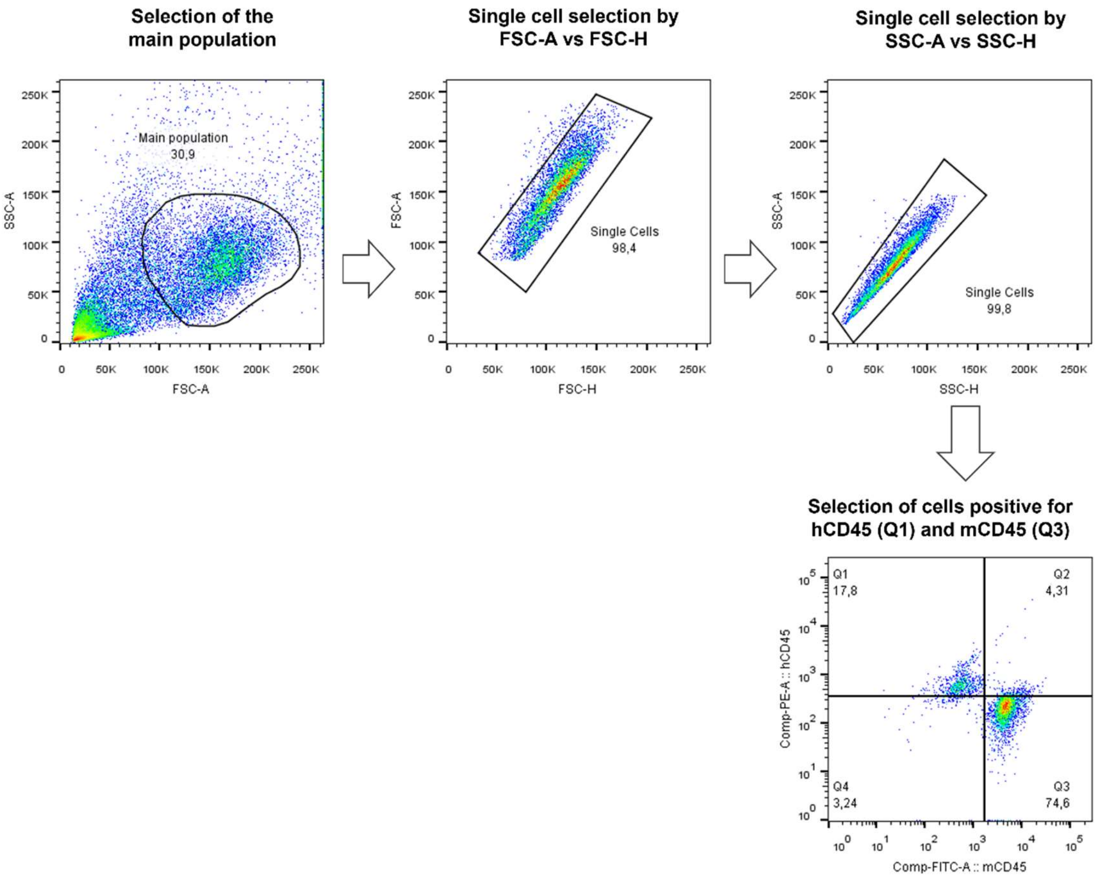

Figure S7. A representative gating strategy was used to select cells positive for mCD45 and hCD45. Dot plots represent peripheral blood sample from NSG mice engrafted with KG-1a cells. FITC-conjugated anti-mouse CD45 (mCD45) and PE-conjugated anti-human CD45 (hCD45) antibodies were used.

Table S1. Cytotoxicity of emetine.

**Cells**	**IC_{50} and 95% CI (μM)**	
	**Emetine**	**DOX**
Hematological cancer cells		
KG-1a	0.94<br>0.49 – 1.79	0.14<br>0.11 – 0.22
HL-60	1.22<br>0.73 – 2.06	0.12<br>0.06 – 0.23
NB4	3.96<br>3.26 – 4.81	0.41<br>0.12 – 1.44
THP-1	0.88<br>0.64 – 1.23	0.23<br>0.14 – 0.40
Jurkat	0.64<br>0.31 – 1.32	0.02<br>0.01 – 1.13
K-562	1.77<br>0.77 – 4.11	1.40<br>0.77 – 2.52
Solid cancer cells		
MCF-7	2.55<br>1.52 – 4.27	1.40<br>0.87 – 2.17

4T1	1.30<br>0.65 – 2.59	1.53<br>1.09 – 2.17
HCT116	0.06<br>0.02 – 0.21	0.56<br>0.40 – 0.81
B16-F10	4.35<br>2.52 – 7.49	0.10<br>0.06 – 0.17
HepG2	4.09<br>2.57 – 6.50	0.44<br>0.31 – 0.58
HSC-3	0.49<br>0.23 – 1.03	0.54<br>0.35 – 0.83
CAL27	0.59<br>0.26 – 1.39	0.10<br>0.02 – 0.40
SCC-9	1.67<br>0.98 – 2.84	1.14<br>0.64 – 2.01
SCC-25	3.22<br>2.18 – 4.75	1.31<br>0.71 – 2.38
Noncancerous cells		
MRC-5	3.79<br>2.70 – 5.31	1.50<br>0.93 – 2.38
BJ	3.74<br>1.93 – 7.22	3.23<br>1.63 – 6.40

PBMC	2.44	1.31
	1.76 – 3.40	0.90 – 1.94

These data were obtained by nonlinear regression from three independent experiments performed in duplicate by the Alamar blue assay after 72 h of incubation. Doxorubicin (DOX) was used as a positive control.

Table S2. Selectivity indexes obtained.

**Cancer cells**	**Noncancerous cells**					
	**MRC-5**		**BJ**		**PBMC**	
	**Emetine**	**DOX**	**Emetine**	**DOX**	**Emetine**	**DOX**
Hematological cancer cells						
KG-1a	4.03	10.71	3.98	23.07	2.60	9.36
HL-60	3.11	12.50	3.07	26.92	2.00	10.92
NB4	0.96	3.66	0.94	7.88	0.62	3.20
THP-1	4.31	6.52	4.25	14.04	2.77	5.70
Jurkat	5.92	75.00	5.84	161.50	3.81	65.50
K-562	2.14	1.07	2.11	2.31	1.38	0.94
Solid cancer cells						
MCF-7	1.49	1.07	1.47	2.31	0.96	0.94
4T1	2.92	0.98	2.88	2.11	1.88	0.86
HCT116	63.17	2.68	62.33	5.77	40.67	2.34
B16-F10	0.87	15.00	0.86	32.30	0.56	13.10
HepG2	0.93	3.41	0.91	7.34	0.60	2.98
HSC-3	7.73	2.78	7.63	5.98	4.98	2.43
CAL27	6.42	15.00	6.34	32.30	4.14	13.10
SC C-9	2.27	1.32	2.24	2.83	1.46	1.15
SC C-25	1.18	1.15	1.16	2.47	0.76	1.00

The data were calculated using the following formula: selectivity index = IC_{50} [noncancerous cells]/IC_{50} [cancer cells].

Table S3. Effect of emetine on the body and relative organ weights of NSG mice with KG-1a cell xenografts.

Parameters	CTL	Emetine
Dose (mg/kg)	-	10 mg/kg
Survival	6/6	6/6
Initial body weight (g)	22.00 ± 0.52	24.83 ± 1.32
Final body weight (g)	20.98 ± 1.15	24.10 ± 1.32
Liver (g/100 g body weight)	5.4 ± 0.42	5.07 ± 0.31
Kidney (g/100 g body weight)	1.22 ± 0.14	1.31 ± 0.17
Heart (g/100 g body weight)	0.47 ± 0.03	0.50 ± 0.04
Lung (g/100 g body weight)	0.81 ± 0.06	0.73 ± 0.07
Spleen (g/100 g body weight)	0.27 ± 0.02	0.19 ± 0.01 *

After engraftment confirmation, the mice were randomly divided into two groups (n=6/per group): a negative control group (5% DMSO) and a group treated with emetine at 10 mg/kg. The treatments were injected into the mice intraperitoneally every day for 2 weeks. The data are presented as the mean ± S.E.M. of 6 animals. * $p < 0.05$ compared to CTL (negative control) by Student's *t* test.

Table S4. The effect of emetine on gene expression in KG-1a cells.

Function/Assay ID	Gene Symbol	Gene Name	RQ	
			CTL	emetine
**NFkB pathway**				
Hs00765730_m1	*NFKB1*	nuclear factor kappa B subunit 1	1.00	10.59
Hs00174517_m1	*NFKB2*	nuclear factor kappa B subunit 2	1.00	64.18
Hs00153283_m1	*NFKBIA*	NFKB inhibitor alpha	1.00	24.37
Hs00182115_m1	*NFKBIB*	NFKB inhibitor beta	1.00	4.05
Hs00153294_m1	*RELA*	RELA proto-oncogene, NF-kB subunit	1.00	4.36
Hs00232399_m1	*RELB*	RELB proto-oncogene, NF-kB subunit	1.00	64.73
**WNT/β-catenin pathway**				
Hs00181051_m1	*APC*	APC, WNT signaling pathway regulator	1.00	4.82
Hs00793391_m1	*CSNK1A1*	casein kinase 1 alpha 1	1.00	4.30
Hs00170025_m1	*CTNNB1*	catenin beta 1	1.00	4.38
Hs00275656_m1	*GSK3B*	glycogen synthase kinase 3 beta	1.00	3.51

Hs00228741_m1	*WNT10A*	Wnt family member 10A	n.d.	n.d.
Hs00559664_m1	*WNT10B*	Wnt family member 10B	1.00	0.05
Hs00257131_m1	*WNT2B*	Wnt family member 2B	n.d.	n.d.
Hs00362452_m1	*WNT6*	Wnt family member 6	n.d.	n.d.
**Hedgehog pathway**				
Hs00368306_m1	*DHH*	desert hedgehog	n.d.	n.d.
Hs00171790_m1	*GLI1*	GLI family zinc finger 1	1.00	1.14
Hs00257977_m1	*GLI2*	GLI family zinc finger 2	n.d.	n.d.
Hs00181117_m1	*PTCH1*	patched 1	1.00	4.27
Hs00179843_m1	*SHH*	sonic hedgehog	n.d.	n.d.
Hs00170665_m1	*SMO*	smoothened, frizzled class receptor	n.d.	n.d.
Hs00171981_m1	*SUFU*	SUFU negative regulator of hedgehog signaling	1.00	2.33
**NOTCH pathway**				
Hs00194509_m1	*DLL1*	delta like canonical Notch ligand 1	1.00	13.19
Hs01085096_m1	*DLL3*	delta like canonical Notch ligand 3	1.00	6.21

Hs00164982_m1	*JAG1*	jagged 1	1.00	1.26
Hs00171432_m1	*JAG2*	jagged 2	1.00	5.12
Hs01062014_m1	*NOTCH1*	notch 1	1.00	2.09
Hs01050702_m1	*NOTCH2*	notch 2	1.00	1.65
**EGFR pathway**				
Hs01099999_m1	*EGF*	epidermal growth factor	1.00	n.d.
Hs01076078_m1	*EGFR*	epidermal growth factor receptor	n.d.	n.d.
Hs00364282_m1	*KRAS*	KRAS proto-oncogene, GTPase	1.00	4.13
Hs01046830_m1	*MAPK1*	mitogen-activated protein kinase 1	1.00	2.57
Hs00234119_m1	*RAF1*	Raf-1 proto-oncogene, serine/threonine kinase	1.00	3.26
Hs00269660_s1	*RHOB*	ras homolog family member B	n.d.	n.d.
**JAK/STAT pathway**				
Hs01026983_m1	*JAK1*	Janus kinase 1	1.00	3.05
Hs01078136_m1	*JAK2*	Janus kinase 2	1.00	4.63
Hs00169663_m1	*JAK3*	Janus kinase 3	n.d.	n.d.
Hs01013989_m1	*STAT1*	signal transducer and activator of transcription 1	1.00	20.56

Hs00374280_m1	*STAT3*	signal transducer and activator of transcription 3	1.00	9.20
Hs00273500_m1	*STAT5B*	signal transducer and activator of transcription 5B	1.00	2.25
Hs00598625_m1	*STAT6*	signal transducer and activator of transcription 6	1.00	3.35
**PI3K/AKT/MTOR pathway**				
Hs00178289_m1	*AKT1*	AKT serine/threonine kinase 1	1.00	0.93
Hs01086102_m1	*AKT2*	AKT serine/threonine kinase 2	1.00	1.21
Hs00234508_m1	*MTOR*	mechanistic target of rapamycin	1.00	2.06
Hs00904054_m1	*PIK3C2A*	phosphatidylinositol-4-phosphate 3-kinase catalytic subunit type 2 alpha	1.00	3.63
Hs00176908_m1	*PIK3C3*	phosphatidylinositol 3-kinase catalytic subunit type 3	1.00	5.10
Hs00907957_m1	*PIK3CA*	phosphatidylinositol-4,5-bisphosphate 3-kinase catalytic subunit alpha	1.00	4.91

Hs02621230_s1	*PTEN*	phosphatase and tensin homolog	1.00	3.62
**TGF-beta/SMAD pathway**				
Hs01054576_m1	*FOXO1*	forkhead box O1	1.00	27.41
Hs00183425_m1	*SMAD2*	SMAD family member 2	1.00	8.50
Hs00929647_m1	*SMAD4*	SMAD family member 4	1.00	2.01
Hs00178696_m1	*SMAD7*	SMAD family member 7	1.00	6.95
Hs00998133_m1	*TGFB1*	transforming growth factor beta 1	1.00	2.62
Hs00234244_m1	*TGFB2*	transforming growth factor beta 2	1.00	3.41
Hs01086000_m1	*TGFB3*	transforming growth factor beta 3	1.00	24.25
**PPAR pathway**				
Hs00947536_m1	*PPARA*	peroxisome proliferator activated receptor alpha	1.00	1.53
Hs04187066_g1	*PPARD*	peroxisome proliferator activated receptor delta	1.00	7.78
Hs01115513_m1	*PPARG*	peroxisome proliferator activated receptor gamma	1.00	n.d.
Hs01016719_m1	*PPARGC1A*	PPARG coactivator 1 alpha	n.d.	n.d.
Hs00991677_m1	*PPARGC1B*	PPARG coactivator 1 beta	1.00	0.38

**Oxidative stress**				
Hs00943350_g1	*GSTP1*	glutathione S-transferase pi 1	1.00	0.64
Hs00178247_m1	*OXSR1*	oxidative stress responsive 1	1.00	1.87
Hs00167309_m1	*SOD2*	superoxide dismutase 2, mitochondrial	1.00	1.93
Hs01555214_g1	*TXN*	thioredoxin	1.00	0.83
Hs00917067_m1	*TXNRD1*	thioredoxin reductase 1	1.00	1.98
**Apoptosis**				
Hs00559441_m1	*APAF1*	apoptotic peptidase activating factor 1	1.00	6.18
Hs00188930_m1	*BAD*	BCL2 associated agonist of cell death	1.00	1.57
Hs00180269_m1	*BAX*	BCL2 associated X, apoptosis regulator	1.00	1.77
Hs99999018_m1	*BCL2*	BCL2, apoptosis regulator	1.00	0.12
Hs00609632_m1	*BID*	BH3 interacting domain death agonist	1.00	4.46
Hs00234387_m1	*CASP3*	caspase 3	1.00	4.00

Hs00169152_m1	*CASP7*	caspase 7	1.00	3.09
Hs00219876_m1	*DIABLO*	diablo IAP-binding mitochondrial protein	1.00	3.22
Hs00538709_m1	*FADD*	Fas associated via death domain	1.00	7.15
Hs00531110_m1	*FAS*	Fas cell surface death receptor	1.00	1.65
Hs00242302_m1	*PARP1*	poly(ADP-ribose) polymerase 1	1.00	0.32
**Autophagy**				
Hs01047860_g1	*ATG12*	autophagy related 12	1.00	2.55
Hs00223937_m1	*ATG3*	autophagy related 3	1.00	2.53
Hs00169468_m1	*ATG5*	autophagy related 5	1.00	1.52
Hs00186838_m1	*BECN1*	beclin 1	1.00	1.60
Hs01076567_g1	*MAP1LC3A*	microtubule associated protein 1 light chain 3 alpha	1.00	n.d.
Hs00797944_s1	*MAP1LC3B*	microtubule associated protein 1 light chain 3 beta	1.00	18.09
**Necroptosis**				
Hs00169407_m1	*RIPK1*	receptor interacting serine/threonine kinase 1	1.00	3.18

Hs01572686_m1	*RIPK2*	receptor interacting serine/threonine kinase 2	1.00	2.46
Hs01011177_g1	*RIPK3*	receptor interacting serine/threonine kinase 3	1.00	3.15
**Epithelial–mesenchymal transition**				
Hs00170423_m1	*CDH1*	cadherin 1	n.d.	n.d.
Hs00195591_m1	*SNAI1*	snail family transcriptional repressor 1	1.00	1.18
Hs00950344_m1	*SNAI2*	snail family transcriptional repressor 2	n.d.	n.d.
Hs01018996_m1	*SNAI3*	snail family transcriptional repressor 3	1.00	2.13
Hs00361186_m1	*TWIST1*	twist family bHLH transcription factor 1	1.00	0.44
Hs00185584_m1	*VIM*	vimentin	1.00	8.81
Hs00232783_m1	*ZEB1*	zinc finger E-box binding homeobox 1	1.00	6.92
Hs00207691_m1	*ZEB2*	zinc finger E-box binding homeobox 2	1.00	19.82

KG-1a cells were treated with 2 μM emetine for 12 h. The negative control (CTL) was treated with the vehicle (0.2% DMSO) used for diluting the test compound. After treatment, total RNA was isolated and reverse transcribed. Gene expression was detected using a TaqMan® array plate 96 plus fast (#4413256). The *GUSB*, *HPRT1* and *GAPDH* genes were used as endogenous genes for normalization. The values represent the relative quantitation (RQ) compared with the calibrator (cells treated with the negative control). The genes were upregulated if RQ ≥ 2 and downregulated if RQ ≤ 0.5. N.d. Not determined.

Table S5. List of cells used.

Cells	Histological type	Species	Source[a,b]
Cancer cell lines			
KG-1a	acute myelogenous leukemia	human	ATCC
HL-60	acute promyelocytic leukemia	human	ATCC
NB4	acute promyelocytic leukemia	human	ATCC
THP-1	monocytic leukemia	human	ATCC
Jurkat	T-cell lymphoid leukemia	human	ATCC
K-562	chronic myelogenous leukemia	human	ATCC
MCF-7	breast adenocarcinoma	human	ATCC
4T1	breast carcinoma	mouse	ATCC
HCT116	colorectal carcinoma	human	ATCC
B16-F10	melanoma	mouse	ATCC
HepG2	hepatocellular carcinoma	human	ATCC
HSC-3	oral squamous cell carcinoma	human	ATCC
CAL 27	oral squamous cell carcinoma	human	ATCC
SCC-9	oral squamous cell carcinoma	human	ATCC

SCC-25	oral squamous cell carcinoma	human	ATCC
Noncancer cell lines			
MRC-5	lung fibroblast	human	ATCC
BJ	foreskin fibroblast	human	ATCC
Mutant and its parental cell lines			
BAD KO SV40 MEF	immortalized mouse embryonic fibroblasts with the BAD gene knocked out	mouse	ATCC
WT SV40 MEF	wild type immortalized embryonic fibroblasts	mouse	ATCC
Primary cells			
PBMC	health peripheral blood mononuclear cells	human	primary cell culture

[a]ATCC denotes the American Type Culture Collection (USA). [b]Primary cell culture of PBMCs was obtained from peripheral blood from healthy donors by a standard Ficoll density protocol. Then, the PBMCs were resuspended in RPMI 1640 or DMEM-F12 supplemented with 20% FBS and 1% antibiotics. The cells were plated at 5×10^5 cells/well. Concanavalin A (10 μg/mL, Sigma–Aldrich) was used as a mitogen to trigger cell division in T lymphocytes and was added at the beginning of the culture. The Research Ethics Committee of the Oswaldo Cruz Foundation (Salvador, Bahia, Brazil) approved the protocol (CAAE 16220713.2.0000.0040).

Table S6. Antibodies used.

Antibody	Fluorochrome	Reactivity	Clone	Catalog number	Manufacturer
Active caspase 3	FITC	Human/Mouse	C92-605	559341	BD Pharmingen™
Cleaved PARP (Asp214)	PE	Human	F21-852	552933	BD Pharmingen™
NF-κB p65 (pS529)	AF488	Human	K108951250	558421	BD Phosflow™
NF-κB p65	PE	Human	14G10A21	653004	BioLegend
CD13	PE-CF594	Human	WM15	562491	BD Horizon™
CD14	PerCP-Cy5	Human	63D3	367110	Biolegend
CD33	BV510	Human	WM53	563257	BD Horizon™
CD34	PE	Human	8G12	348057	BD™
CD38	BV421	Human	HIT2	562444	BD Horizon™
CD97	APC	Human	MEM-180	MA5-16825	Thermo Fisher
CD99	PE	Human	3B2-TA8	371305	Biolegend

CD123	BV605	Human	7G3	564197	BD Horizon™
IgG1, κ Isotype Control	PE	-	MOPC-21	556650	BD Pharmingen™
CD45 (hCD45)	PE	Human	2D1	368509	BioLegend
CD45 (mCD45)	FITC	Mouse	30-F11	103107	BioLegend
